# Supplementary figures and images for: Global Wheat Head Detection (GWHD) Dataset: A Large and Diverse Dataset of High-Resolution RGB-Labelled Images to Develop and Benchmark Wheat Head Detection Methods
Source: Plant Phenomics. 2020 Aug 20;2020:3521852. doi: 10.34133/2020/3521852 (PMC7706323; doi:10.34133/2020/3521852)

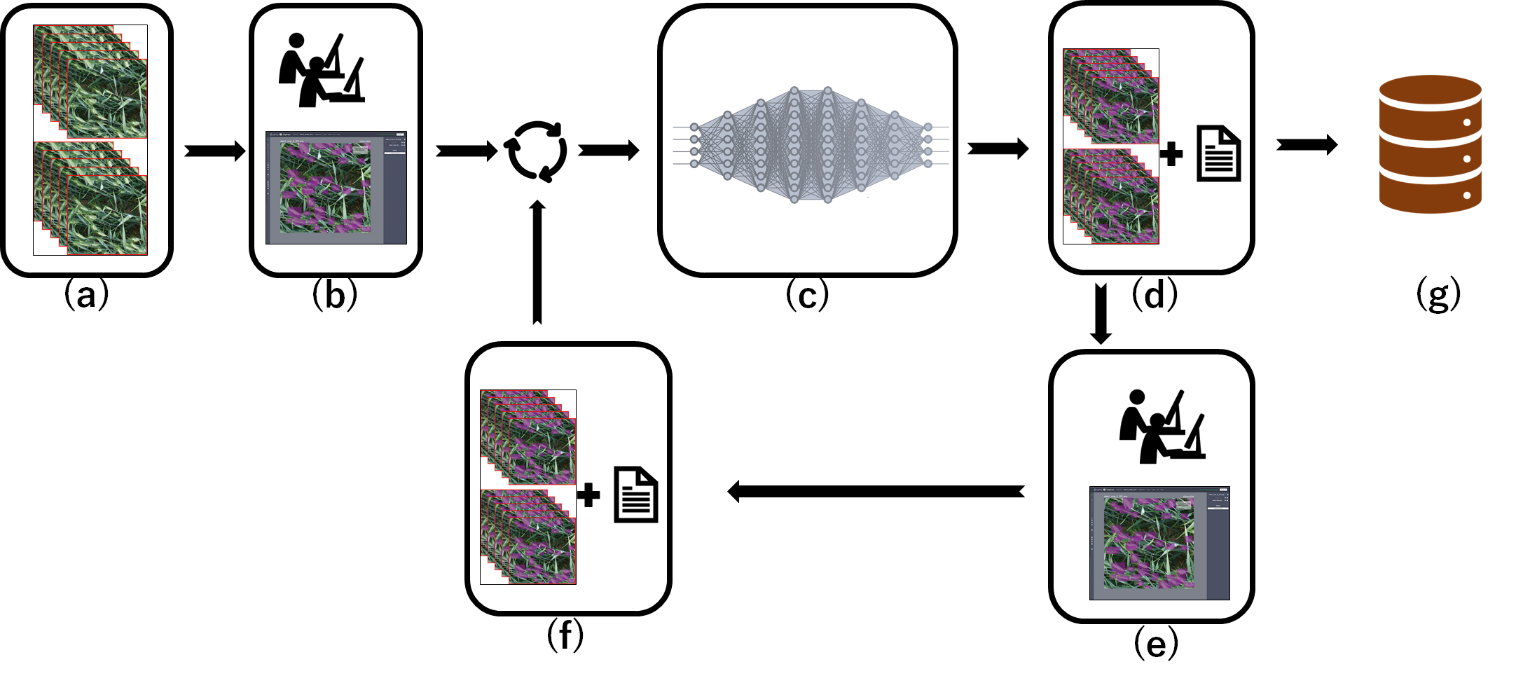

Supplement: Supplementary Materials — Figure S1: the proposed “weakly supervised deep learning framework” to prelabel images efficiently. Figure S2: epoch-wise results (RMSE, rRMSE, R2, mAP@0.5) of Faster-RCNN baseline with ResNet34 and ResNet50. The best model is obtained at epoch 3 for both backbones. [file 3521852.f1.zip › figure_S1.png]

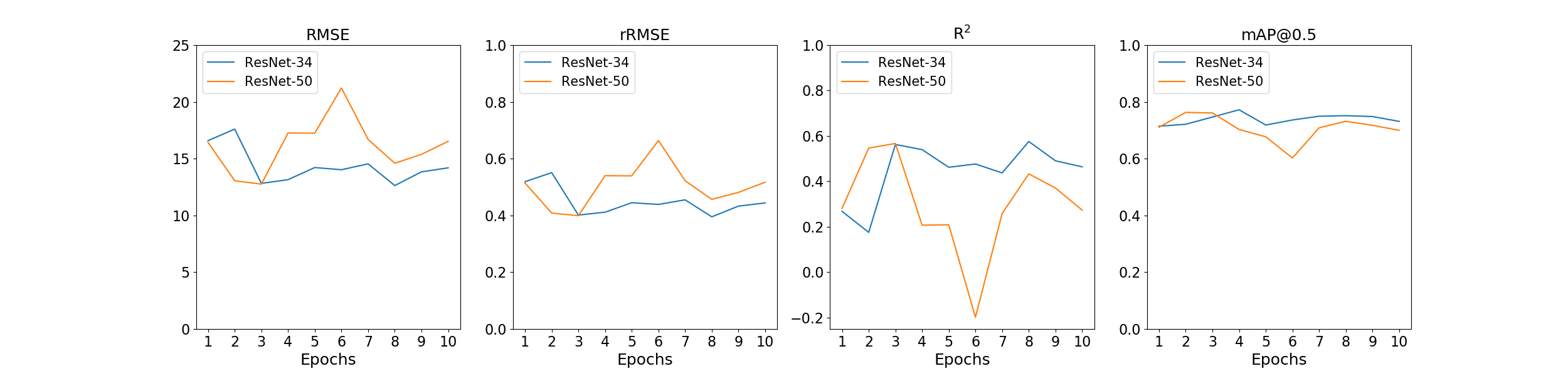

Supplement: Supplementary Materials — Figure S1: the proposed “weakly supervised deep learning framework” to prelabel images efficiently. Figure S2: epoch-wise results (RMSE, rRMSE, R2, mAP@0.5) of Faster-RCNN baseline with ResNet34 and ResNet50. The best model is obtained at epoch 3 for both backbones. [file 3521852.f1.zip › figure_S2.png]
